# Anterior-Segment Optical Coherence Tomography Unlocks Novel Perspectives: Lacking Iris Anterior Limiting Layer Signal in Uveitis

**DOI:** 10.7759/cureus.51872

**Published:** 2024-01-08

**Authors:** Joobin Khadamy

**Affiliations:** 1 Ophthalmology, University Hospital of Umeå, Umeå, SWE; 2 Ophthalmology, Skellefteå Eye Clinic, Skellefteå, SWE

**Keywords:** anterior-segment oct, uveitic changes, ophthalmic diagnostics, iris abnormalities and koeppe nodule, ocular imaging, chronic uveitis, as-oct findings, intraocular inflammation, neurosarcoidosis, uveitis

## Abstract

Chronic uveitis, a challenging intraocular inflammatory condition, presents complexities in diagnosis and management due to its diverse etiologies and manifestations. Anterior-segment optical coherence tomography (AS-OCT) has emerged as a pivotal tool in evaluating uveitis, offering high-resolution imaging of anterior segment structures. We present the case of a 49-year-old man diagnosed with neurosarcoidosis and chronic intermediate uveitis, where AS-OCT revealed unique findings. Clinical examination identified a Koeppe nodule. AS-OCT evaluation unveiled hyperreflectivity in the iris stroma and the adjacent nodule. Notably, AS-OCT documented the absence of the hyperreflective anterior limiting layer signal, a novel observation in uveitis assessment. This unprecedented finding underscores the significance of AS-OCT in elucidating uveitis pathophysiology and emphasizes its potential in refining diagnostic and therapeutic strategies for this complex ocular condition.

## Introduction

The comprehensive nature of uveitis, marked by diverse etiologies and presentations, presents diagnostic and therapeutic challenges rooted in its complex pathogenesis involving immune dysregulation, infectious agents, and systemic diseases. Koeppe nodules, frequently seen during clinical examinations, especially in cases of granulomatous or occasionally non-granulomatous uveitis, appear as small yellowish-white nodules along the pupillary edge of the iris. Information regarding the response of these nodules to treatment or over time, as well as their prognostic significance, is scarce in existing literature [1].

Traditionally, diagnostic approaches have relied on clinical examination, imaging, and laboratory investigations, yet the introduction of anterior-segment optical coherence tomography (AS-OCT) has transformed our ability to visualize intraocular structures precisely. AS-OCT, a non-invasive imaging modality, proves pivotal in uveitis evaluation, offering high-resolution cross-sectional images that reveal subtle structural changes in the cornea, anterior chamber, iris, and ciliary body. This technology enhances diagnostic accuracy and facilitates disease monitoring by identifying specific inflammatory features, such as cellular infiltration and tissue alterations [2]. This report showcases a groundbreaking discovery in a patient with sarcoidosis, shedding light on potential advancements in understanding the pathogenesis of uveitis.

## Case presentation

A 49-year-old male with a history of neurosarcoidosis and chronic intermediate uveitis presented without any documented history of trauma or ocular surgery. The diagnosis, established a decade ago, was supported by MRI findings and systemic sarcoidosis evidence derived from histological examination via bronchoscopy involving endobronchial and transbronchial lung biopsy. Additionally, chest imaging and the observation of snowball-like vitreous opacities in the eyes substantiated the diagnosis. Treatment encompassed methotrexate (10 mg orally once a week), low-dose oral corticosteroids (5 mg orally daily), and intermittent corticosteroid eye drops during relapses. The present ocular examination revealed aged vitreous snowballs alongside low-grade cells (+0.5 in SUN grading) and faint flare (SUN grading) in the anterior chamber. Gonioscopy findings were unremarkable.

**Figure 1:**
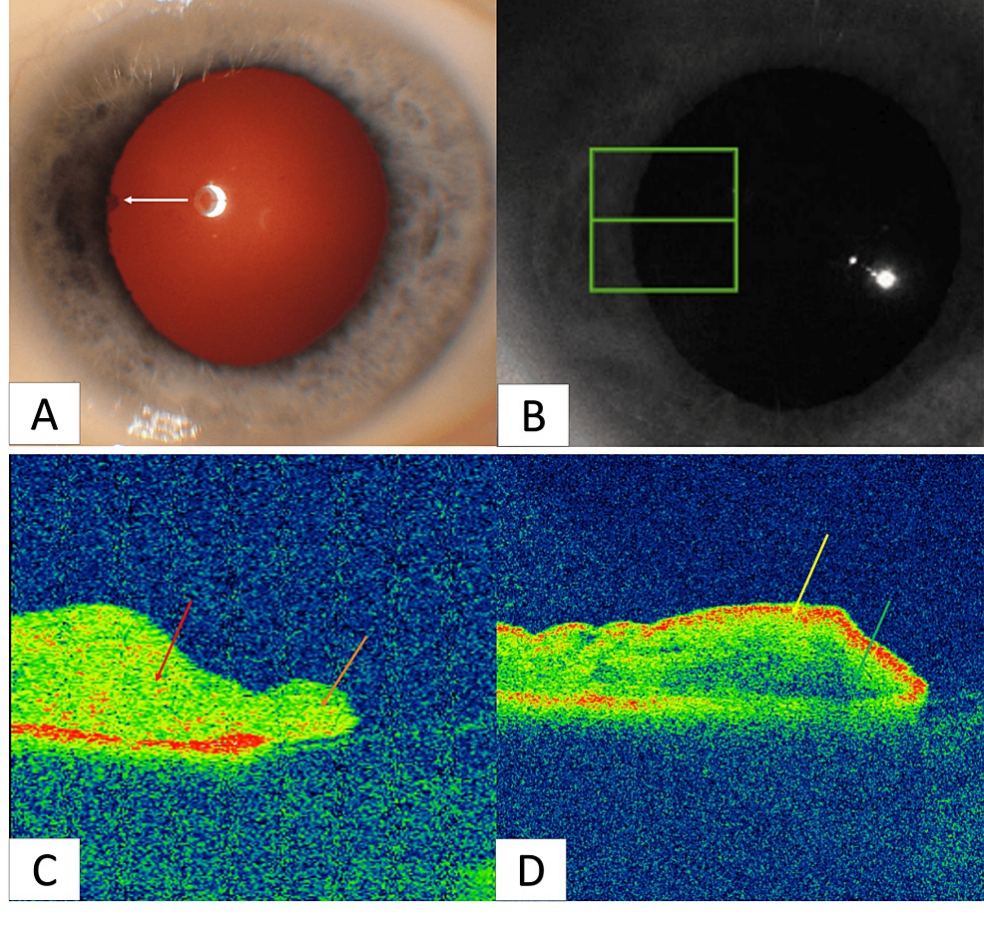
AS-OCT reveals the absence of iris anterior limiting layer signal and hyperreflectivity in uveitic iris stroma and Koeppe nodule. A: Koeppe nodule observed on iris examination (white arrow). B: Infrared image indicating the scan position. C: AS-OCT B-scan displaying hyperreflectivity in the iris stroma (red arrow) and adjacent nodule (orange arrow), implying potential inflammatory infiltration. Notably, the absence of a hyperreflective anterior limiting layer signal contrasts with a normal iris scan. D: Normal iris scan revealing a hyperreflective anterior limiting layer (yellow arrow) and lower reflectivity in the iris stroma (green arrow). AS-OCT: anterior-segment optical coherence tomography

Clinical assessment identified a Koeppe nodule on iris examination, prompting investigation with a DRI OCT Triton™ equipped with an AS-OCT module. AS-OCT imaging revealed hyperreflectivity within the iris stroma and the adjoining nodule, suggestive of potential inflammatory infiltration. Notably, comparison with a standard iris scan highlighted the striking absence of the hyperreflective anterior limiting layer signal in the uveitic eye, representing a novel observation in this context (Figure 1).

## Discussion

The revelation of the missing hyperreflective anterior limiting layer signal in a uveitis case observed via AS-OCT signifies a notable discovery, suggesting alterations in this layer that might facilitate inflammatory cell infiltration beyond iris borders, leading to Koeppe nodules. This finding necessitates further confirmation through subsequent studies.

Research on iris change in uveitis is notably scarce, lacking correspondence between observed iris layers via AS-OCT and histopathological sections. Exploration is needed to determine whether these alterations involve biological, chemical, or structural shifts. Connecting these findings to histopathological observations is crucial for understanding the pathophysiology of uveitis. It is uncertain if these changes are disease-specific or exclusive to granulomatous uveitis (e.g., sarcoidosis) with Koeppe nodules. Determining whether alterations in the anterior limiting layer precede or follow Koeppe nodule development is crucial. Investigating whether these changes reverse after inflammation control and whether their detection indicates inadequate suppression or holds prognostic value warrants further exploration. Although the clinical significance of anterior limiting layer changes remains unclear, it potentially impacts the understanding of iris tumors and uveitis management.

The utilization of AS-OCT to study the iris in uveitis presents a unique opportunity for real-time observation of inflammatory changes comparable to histopathological studies. However, its limited availability in low-income countries might hinder its widespread use in routine uveitis management. Nonetheless, where accessible, it offers novel insights into understanding the complexities of this disease. The potential of AS-OCT in routine uveitis assessment and follow-up holds promise. Its ability to evaluate morphology, layers, and thickness of the iris [3]; presence and grading of the cell and flare in the anterior chamber (AC) [4,5]; peripheral anterior and pupillary synechiae [6]; and morphology and counts of the keratic precipitates [7] may profoundly influence uveitis management [2,8].

Despite uveitis frequently co-occurring with systemic rheumatic diseases, the severity or presence of uveitis does not consistently align with the activity level of the associated systemic disease [1]. However, utilizing AS-OCT of the iris to examine eye inflammation offers a real-time, dynamic in vivo histopathological insight into inflammatory changes occurring in the body, shared between both uveitis and systemic rheumatic conditions, ultimately enriching our comprehension of these complex inflammatory disorders.

Overall, the incorporation of AS-OCT revolutionizes uveitis evaluation, providing substantial insights into anatomical changes, pathogenesis, and comprehensive uveitis care. Ongoing research aims to enhance its usefulness, potentially transforming uveitis diagnostics and treatment monitoring.

## Conclusions

This case underscores the distinctive AS-OCT revelations within chronic uveitis, particularly the absence of the hyperreflective anterior limiting layer signal. It accentuates the promising utility of AS-OCT as a crucial asset in uveitis assessment. Continued research and the aggregation of similar cases hold the potential to reshape our comprehension of uveitis, paving the way for refined diagnostic and therapeutic strategies in managing uveitic conditions. The unique findings unveiled through AS-OCT in this case set the stage for a deeper exploration of the role of this imaging modality in elucidating the intricacies of uveitis, potentially revolutionizing its management and clinical approach.
